# Effects of ketamine on fear memory extinction: a review of preclinical literature

**DOI:** 10.3389/fnins.2025.1546460

**Published:** 2025-04-30

**Authors:** Martin Boese, Rina Berman, Kennett Radford, Luke R. Johnson, Kwang Choi

**Affiliations:** ^1^Daniel K. Inouye Graduate School of Nursing, Uniformed Services University, Bethesda, MD, United States; ^2^Center for the Study of Traumatic Stress, Uniformed Services University, Bethesda, MD, United States; ^3^Walter Reed National Military Medical Center, Bethesda, MD, United States; ^4^School of Psychological Sciences, College of Health and Medicine, University of Tasmania, Hobart, TAS, Australia; ^5^Department of Psychiatry, Uniformed Services University, Bethesda, MD, United States

**Keywords:** ketamine, fear memory, posttraumatic stress disorder (PTSD), fear extinction, cognitive behavioral therapy (CBT), review, translational research, molecular mechanism

## Abstract

**Introduction:**

Ketamine, a multimodal dissociative anesthetic, is widely used as a trauma analgesic in emergency situations. Ketamine is also used to treat psychiatric disorders due to its broad application potential, including treatment-resistant major depression. However, its impacts on the development of post-traumatic stress disorder (PTSD) and its potential as a treatment for PTSD are controversial. PTSD is marked by persistent and intrusive memories of traumatic event(s) and re-experiencing of the traumatic memories when exposed to trauma-related stimuli. Individuals with PTSD are often treated with prolonged exposure therapy (PE), in which they are gradually exposed to stimuli that remind them of the previous traumatic memory. If successful, they may learn that the previously traumatic stimuli are no longer threatening, a process known as fear extinction. Although fear extinction can be studied in laboratory animals, previous preclinical literature on the effects of ketamine on fear extinction has been inconsistent.

**Methods:**

Thus, we summarized the existing preclinical literature examining effects of ketamine on fear extinction and its potential molecular mechanisms.

**Results:**

Studies found that ketamine may enhance, impair, have no effect, or have mixed effects on fear extinction. These discrepancies may be attributed to differences in dosage, route, and timing of ketamine administration.

**Discussion:**

We conclude the review with recommendations for future research on ketamine and PTSD such as the inclusion of more female subjects, clinically relevant doses and routes of ketamine administration, and more comprehensive behavioral assays that are relevant to PTSD in humans to enhance translation between preclinical and clinical research.

## Posttraumatic stress disorder

Posttraumatic stress disorder (PTSD) is a psychiatric disorder involving traumatic events such as stress, injury, sexual assault, and military combat (Qi et al., 2016; Wynn et al., 2017). Symptoms of PTSD include intrusions; avoidance of stimuli related to the trauma; negative alterations in cognitions and mood; and marked changes in arousal and reactivity, including hypervigilance, as listed in the Diagnostic and Statistical Manual of Mental Disorders, Fifth Edition (DSM-5) (American Psychiatric Association, 2022). Therefore, PTSD can be a significant threat and burden to the quality of life in affected individuals.

Approximately 7% of the general population may experience PTSD in their lifetime, and ~3.5% of people experience PTSD symptoms in the U.S. annually (Goldstein et al., 2016). Although exposure to trauma is common in the general population, only a sub-group of those affected individuals will develop PTSD. There are many types of trauma, such as those related to war or combat; sexual violence; physical violence; death of a significant other; accidents; or life-threatening events, which are heterogeneous in their contributions to PTSD risk (Kessler et al., 2017; Liu et al., 2017). A conditional PTSD risk of 4% after exposure to any one trauma was reported (Kessler et al., 2017; Liu et al., 2017).

Currently, PTSD is difficult to diagnose, prevent, and treat among individuals with traumatic events. Standard treatment for PTSD involves pharmacological, cognitive, and behavioral therapies (Raut et al., 2022a), although psychotherapy is often considered a first-line treatment with pharmacotherapy a second-line treatment (Merians et al., 2023). Only two selective serotonin reuptake inhibitors (SSRIs), sertraline and paroxetine, are FDA-approved for the treatment of PTSD in the U.S., although other antidepressant drugs are used off-label (Singewald et al., 2023). Yet, the response rate for pharmacotherapy alone remains poor, as only 59% of patients may respond to antidepressant treatment (Stein et al., 2006). Pharmacological treatment of PTSD is commonly combined with psychotherapy, to include mindfulness-based treatments; prolonged exposure therapy (PE), where a patient gradually confronts feelings, thoughts, and situations related to the trauma and cognitive processing therapy (CPT), where a patient is made to challenge and modify unhelpful thoughts related to the trauma (Merians et al., 2023; Boyd et al., 2018; McLean et al., 2021). It is worth noting that these therapies may have high dropout rates due to logistical reasons, intensity of side-effects, severity of PTSD symptoms, and other factors (Najavits, 2015). Limited efficacy of current treatments and comorbidities with PTSD such as major depressive disorder, anxiety disorder, and substance use disorders (Wynn et al., 2017; Raut et al., 2022b) contribute to high rates of psychotropic polypharmacy in military and veteran populations (Raut et al., 2024). Therefore, more evidence-based diagnosis and prediction of PTSD and improved therapeutic options with pharmacotherapy and CBT would benefit individuals suffering from PTSD.

### Ketamine and PTSD

Ketamine, a non-competitive N-methyl-D-aspartate (NMDA) glutamate receptor antagonist, produces sedation, analgesia, anesthesia, and psychiatric effects in a dose-dependent manner (Corlett et al., 2016; McMillan and Muthukumaraswamy, 2020; Mion and Villevieille, 2013). Ketamine has been shown to be effective in treatment-resistant major depression (TRD) (Berman et al., 2000; Glue et al., 2017; Riggs et al., 2021), as well as effective as an intervention in suicidal ideation (Phillips et al., 2020). Intranasal S-ketamine has been approved by the FDA for management of TRD, and off-label racemic ketamine infusion has demonstrated efficacy in TRD (Swainson et al., 2019; Terao et al., 2024; Loo et al., 2023; Nicolini et al., 2023). Furthermore, the utility of ketamine in psychiatry may extend beyond the treatment of major depression and may be beneficial for treatment of PTSD. For instance, clinical studies have demonstrated that intravenous ketamine infusions are effective in reducing PTSD symptoms in affected individuals (Albott et al., 2018; Feder et al., 2014; Ross et al., 2019). In a retrospective review, U.S. military personnel who were treated with pre-hospital ketamine had significantly lower odds of developing PTSD symptoms (Melcer et al., 2022). Among U.S. service members receiving treatment for burns, PTSD prevalence was 27% in patients receiving ketamine compared to 46% in those not receiving ketamine (McGhee et al., 2008).

However, not all studies found the beneficial effects of ketamine on PTSD symptoms, making the therapeutic effects of ketamine on PTSD controversial. For example, Schonenberg et al. (2008) found increased PTSD symptoms among accident victims who were treated with S-ketamine. Additionally, ketamine had no effects on the development of PTSD or PTSD symptoms in a retrospective study of war-injured service members between 2010 to 2012 (Mion et al., 2017). McGhee et al. (2014) found no benefit of ketamine on PTSD development in burned service members. Another study reported no significant effects of ketamine on PTSD symptoms, although the authors observed improvement in comorbid depression symptoms in service members (Abdallah et al., 2022). In a review article discussing differences in PTSD between civilian and military populations (Fremont et al., 2023), a single dose intravenous (IV) ketamine administration facilitated rapid reduction of PTSD symptoms. Repeated IV ketamine administration also improved PTSD symptoms compared to midazolam in individuals with PTSD. However, in a veteran and military population, repeated IV ketamine did not significantly reduce PTSD symptoms, possibly due to types of trauma and gender differences that may dictate variable responses to ketamine treatment. In a recent review and meta-analysis of 10 clinical studies of ketamine on PTSD (Almeida et al., 2024), four studies investigated the use of ketamine in conjunction with mindfulness-related psychotherapy (Pradhan et al., 2018, 2017) and PE therapy (Shiroma et al., 2020; Harpaz-Rotem, 2022). Although there is variability between studies, ketamine demonstrated significant improvements in PTSD symptoms. Previous preclinical and clinical studies investigated the effects of D-cycloserine, an NMDA receptor partial agonist, on fear extinction. While some studies demonstrated an enhancement of fear extinction learning when given within an hour of the learning session, overall the results are mixed due to variability of timing in drug administration (Grillon, 2009; Davis, 2011; Norberg et al., 2008). Given the inconsistent findings from the previous studies on the impact of ketamine on PTSD and fear memory, a more systematic approach of investigating this topic is urgently needed.

**Figure 1:**
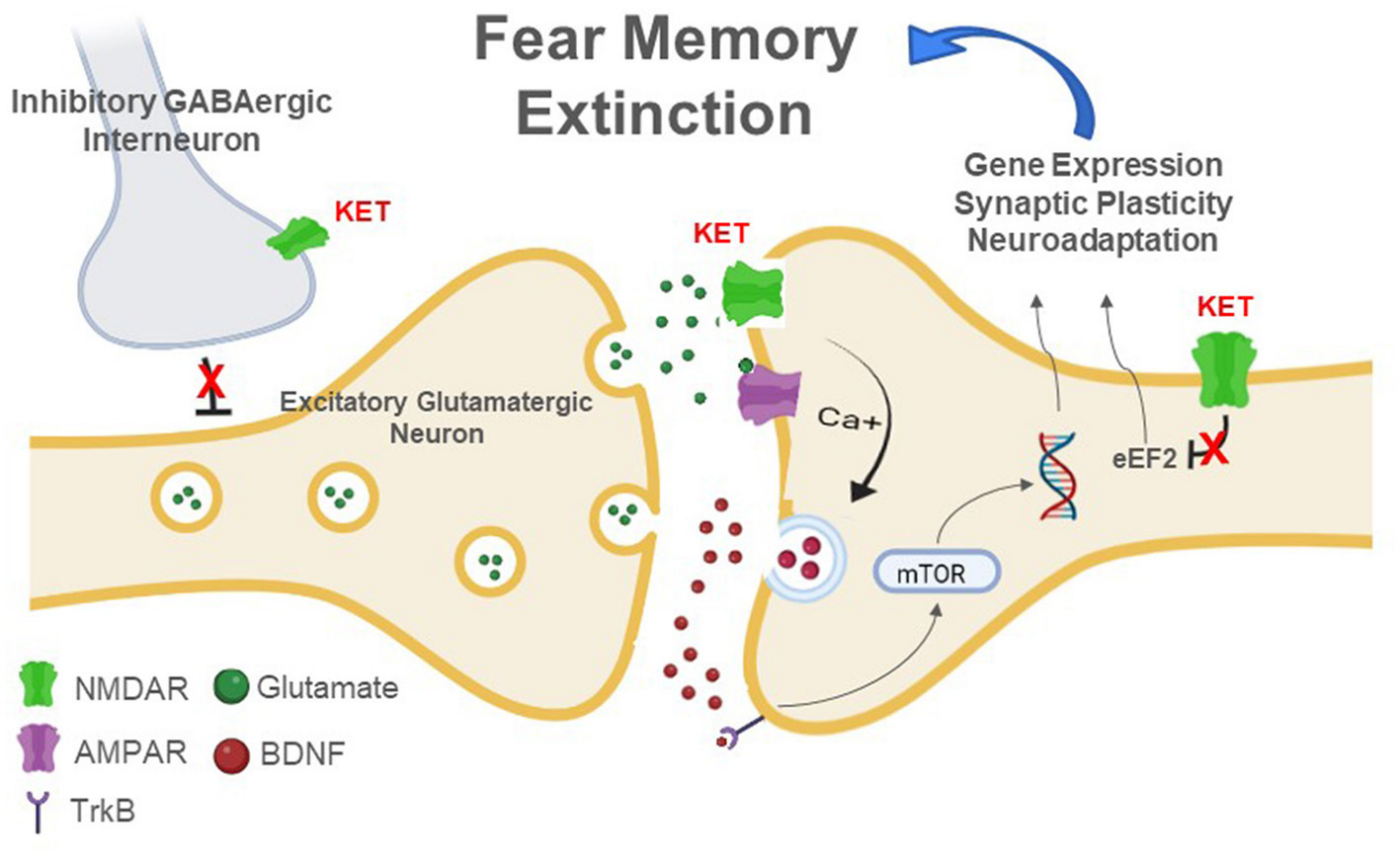
Molecular mechanisms of ketamine on fear memory extinction. GABA, gamma-aminobutyric acid; NMDAR, N-methyl-D-aspartate receptor; AMPAR, α-amino-3-hydroxy-5-methyl-4-isoxazolepropionic acid receptor; TrkB, tyrosine receptor kinase B; BDNF, brain-derived neurotrophic factor; mTOR, mammalian/mechanistic target of rapamycin; Ca++, calcium; eEF2, eukaryotic translation elongation factor 2.

A comprehensive review article summarized existing and novel pharmacological targets in drug development for anxiety-related disorders including PTSD (Sartori and Singewald, 2019). They discussed several potential molecular mechanisms and signaling pathways of ketamine on anxiety disorders and PTSD, which have previously been implicated in ketamine's effects on rodent fear memory (Choi et al., 2020; Asim et al., 2021; Glavonic et al., 2022). Subanesthetic doses of ketamine may inhibit NMDA glutamate receptors on gamma-aminobutyric acid (GABA) inhibitory interneurons (Zanos and Gould, 2018), leading to a paradoxical increase in glutamate release in the brain (Moghaddam et al., 1997). Glutamate activates postsynaptic α-amino-3-hydroxy-5-methyl-4-isoxazolepropionic acid (AMPA) receptors, resulting in increased excitability of neurons (Aleksandrova and Phillips, 2021; Aleksandrova et al., 2017). In turn, AMPA receptor activation potentiates the release of brain-derived neurotrophic factor (BDNF), which upon binding to tyrosine receptor kinase B (TrkB) potentiates the downstream mammalian/mechanistic target of rapamycin (mTOR) pathway to influence synaptic protein synthesis (Aleksandrova and Phillips, 2021; Aleksandrova et al., 2017; Glavonic et al., 2024). Ketamine also potentiates the trafficking of AMPA receptors to the postsynaptic membrane (Aleksandrova et al., 2017; Glavonic et al., 2024), which is critical for long-term potentiation (LTP) and the strengthening of synapses underlying learning and memory (Diaz-Alonso and Nicoll, 2021). Potential signaling pathways for ketamine in the context of fear memory extinction are described in Figure 1. In summary, ketamine binds to NMDA glutamate receptors on GABAergic interneurons to disinhibit glutamate release. This increases excitatory neurotransmission and downstream signaling pathways including BDNF and mTOR in the neuron. These processes will contribute to synaptic plasticity and neuroadaptations in the crucial brain regions that are involved in fear learning and fear memory.

### Fear memory extinction

**Figure 2:**
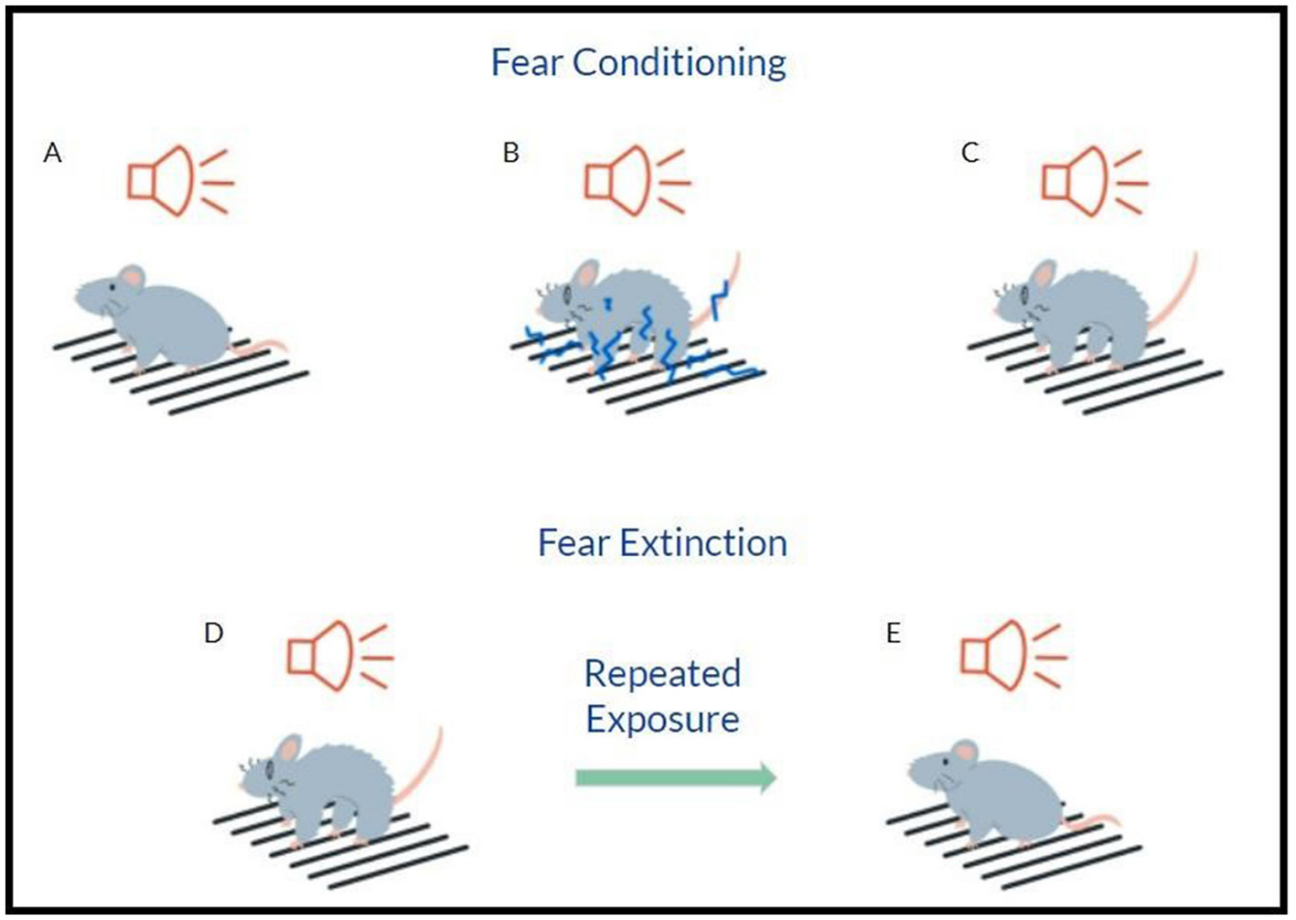
Auditory fear conditioning in rodents. **(A)** Prior to fear conditioning, an animal does not respond to a neutral conditioned stimulus (CS). **(B)** CS is paired with a noxious unconditioned stimulus, such as foot shock. **(C)** Following fear conditioning, the animal recalls fearful memory and displays fear behavior when presented with CS. **(D, E)** Fear memory extinction: after repeated, unreinforced exposures to the CS, the animal learns that the CS no longer represents a threat, and fear memory behaviors are reduced or eliminated.

Preclinical PTSD models allow researchers to investigate the effects of ketamine on PTSD-like behaviors in animals and provide further insight into the mechanisms of PTSD. As PTSD involves recurrent and intrusive traumatic memories, reducing or normalizing traumatic memories in animals may translate to the improvement of PTSD in humans. Thus, the fear memory extinction paradigm in rodents may serve as a model of one of the symptoms of PTSD and also cognitive and behavioral therapy in humans. Fear memory extinction occurs sometime after fear conditioning/learning, and multiple brain regions such as the amygdala, hippocampus, and prefrontal cortex (PFC) are involved in the process (Bergstrom et al., 2011; Jacques et al., 2019; Schafe and LeDoux, 2000). Fear conditioning is accomplished by exposing an animal to an initially neutral environment (context) or a cue such as a light or an auditory tone (Figure 2A). This conditioned stimulus (CS) is paired with an aversive unconditioned stimulus (US), often an electric footshock (Figure 2B). The animals rapidly learn the association between CS and US, demonstrating a fear response such as freezing when they are re-exposed to the CS without US (Figure 2C) (Bergstrom et al., 2011). After memory consolidation, which is stabilized and encoded for long-term storage, fear memory can be retrieved and undergo reconsolidation and/or extinction (Monfils et al., 2009; Schiller et al., 2010). Thus, fear memory extinction represents new learning rather than simple forgetting (Jacques et al., 2019; Milad and Quirk, 2002, 2012; Morgan and LeDoux, 1995) by repeated exposure to the CS or fear-associated context in the absence of the US (Figure 2D). The animals gradually learn that the CS is no longer associated with the US (electric footshock), and therefore, the previously learned fear behavior diminishes (Figure 2E) (Milad and Quirk, 2002, 2012; Salinas-Hernandez and Duvarci, 2021). Fear extinction is a form of safety learning, involving the formation of an inhibitory memory that competes with the original fear memory. This is particularly important in the context of PTSD; previously neutral stimuli become aversive when associated with the traumatic event(s), and exposure therapy can help individuals overcome PTSD.

Though fear extinction in rodents is often defined as a reduction of freezing behavior after repeated exposure to a CS, there are different ways to assess fear extinction in animals. For instance, decreased freezing in a single extinction session (within-session extinction) is a measure of successful fear extinction acquisition. Fear extinction can also be defined as preventing the return of fear upon exposure to the CS in subsequent sessions (between-session extinction). Usually, an extinction retention test utilizes fewer CS presentations so as not to induce further extinction learning. Fear extinction retention tests can be manipulated to induce a relapse of extinction with a function of time (e.g. spontaneous recovery); re-exposure to the aversive context (renewal); or re-exposure to the CS-US pairing (reinstatement) (Jacques et al., 2019; Milad and Quirk, 2002, 2012; Wotjak, 2019). The rationale behind the previous ketamine studies is that ketamine administration may improve fear extinction learning in a preclinical model and therefore ketamine may possess therapeutic potential for its use in PTSD. The current review discusses preclinical studies utilizing different fear extinction paradigms in rodents to assess the impacts of ketamine administration on fear extinction.

## Purpose and hypothesis

The purpose of this review is to examine the preclinical literature on the role of ketamine in altering fear memory extinction in rodents. Based on the efficacy of ketamine in rapidly reducing treatment-resistant depressive symptoms and previous clinical investigations on the efficacy of ketamine administration on PTSD symptoms, we hypothesized that ketamine may produce beneficial effects on PTSD-like behaviors in the context of specific timing, route, and doses of ketamine administration.

## Methods

**Figure 3:**
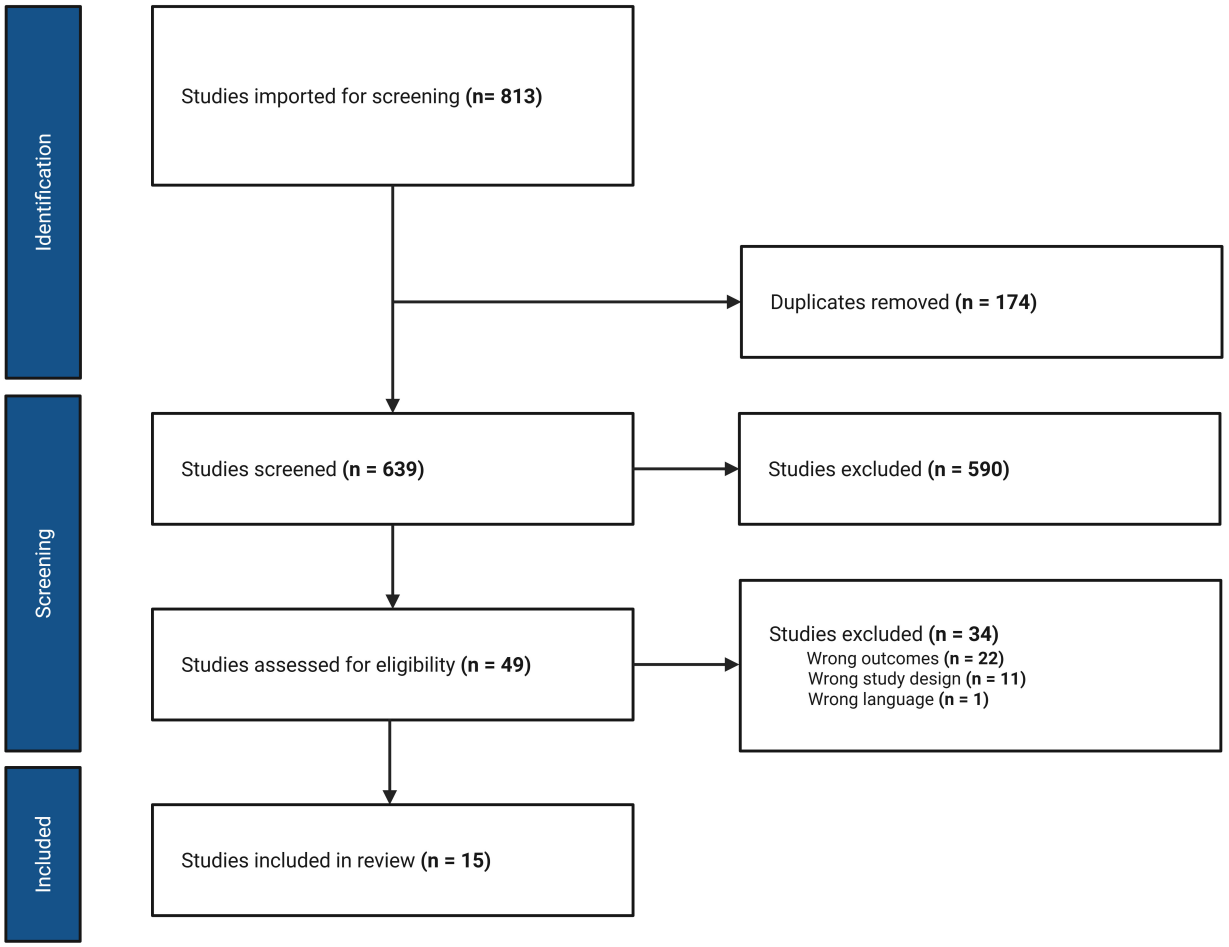
PRISMA flow diagram for literature search and review.

PUBMED and EMBASE databases were searched from inception to June 2024. Keywords included in the initial search were “ketamine” and “fear memory” or “fear memory extinction.” Boolean logic was used to pair the terms, further refining results. Due to limited results, an additional search using “ketamine” and “PTSD” was employed. Inclusion criteria were a preclinical model using rodents, an experimental design (no review articles), and English language articles. The Covidence© online systematic review software (Covidence, Melbourne, Australia) was used to sort and manage the literature search results. Briefly, 813 articles were imported for title and abstract screening, including 809 from database searches and four from a manual literature search of bibliographies, and 174 duplicate titles were removed. Of the remaining 639 articles, 590 were found irrelevant, and 49 were selected for full-text review. Thirty-four articles were removed from consideration, mostly because fear memory extinction was not included as a part of the study design. Fifteen articles met all criteria and they were included in the literature review. These accepted studies spanned from 2015 to 2024. Two independent investigators conducted literature search and data extraction. A Preferred Reporting Items for Systematic reviews and Meta-Analyses (PRISMA) flow diagram detailing the literature search strategy is shown in Figure 3.

## Results

Overall, 15 preclinical research articles were identified which included the effects of ketamine on fear memory extinction in rodents. All articles identified in this review utilized racemic ketamine rather than enantiomers. The majority of the studies (nine out of 15) used rats (6: Sprague Dawley rats, 1: Lister Hooded rats, 1: Long-Evans rats, and 1: Wistar rats), and the remaining six studies used mice (5: C57BL/6 mice and 1: 129S6/SvEvTac mice). The overall findings were inconsistent and those can be summarized to three groups: (1) ketamine enhanced fear memory extinction; (2) ketamine impaired fear memory extinction; (3) ketamine had no effects or mixed results on fear extinction.

### Ketamine enhanced fear memory extinction

Nine studies reported that ketamine administration enhanced fear memory extinction as summarized in Table 1. All of the studies used subanesthetic doses of ketamine, ranging from 0.625 to 30 mg/kg with an intraperitoneal (IP) route of ketamine administration. These effects were noted when ketamine was administered after fear conditioning, with the notable exceptions of two studies (McGowan et al., 2017; Ryan et al., 2022), which found enhanced fear memory extinction when ketamine was given 1 week before fear conditioning.

**Table 1:** Summary of literature that demonstrates enhanced fear extinction with ketamine.

**Study**	**Sex, species, strain, age**	**Dose, route, timing**		**Type of fear/extinction**	**Effect on fear/extinction**	**Notes**
Kulyk et al., 2017	Male, Rats, Long-Evans, Adult	10 mg/kg IP	60 min before FC	-	-	
			Immediately after FC	Early extinction learning	Enhanced
			60 min before extinction training	Early extinction learning	Enhanced
Girgenti et al., 2017	Male, Rats, S-D, 7–9 weeks	10 mg/kg IP, 24 h after FC		Within-session extinction Long-term fear expression	No effect Reduced	Ketamine increased phosphorylation of mTORC1 signaling and c-Fos in mPFC; extinction partially nullified by AMPA receptor inhibitor NBQX
Ju et al., 2017	Male, Mice, C57BL/6, 7–8 weeks	Long-term ketamine (0.625, 1.25, 2.5 mg/kg IP), given daily for 22 days starting after FC		Within-session extinction Long-term fear expression	No effect Reduced	Long-term ketamine (1.25 and 2.5 mg/kg) enhanced BDNF mRNA levels, downregulated methylation of BDNF exon IV, and decreased DNA methyltransferases in the mPFC and hippocampus
		Short-term (5, 10, 20 mg/kg IP) given 2 h prior to extinction training (days 15 and 16)		–	–	
McGowan et al., 2017	Male, Mice, 129S6/SvEvTac, 8 weeks	30 mg/kg IP	1 month, day, or h before CFC	–	-	
		1 week before CFC	Fear expression	Reduced		
Wei et al., 2020	Male, Mice, C57BL/6J, 4 weeks	10 mg/kg IP, 1 h prior to extinction sessions		Within-session extinction	Enhanced on third and fourth sessions	In vitro application of ketamine decreased LTP and PPF in the CA1
Sala et al., 2022	Male, Rats, S-D, Adult	10 mg/kg IP, 6 h after FC		Within-session extinction	Enhanced	Ketamine reversed dendritic atrophy and abnormal glutamate release in the PL-PFC
Ryan et al., 2022	Male, Mice, C57BL/6N, Adult	30 mg/kg IP, 1 week before FC		Between-session extinction	Enhanced extinction to CS-after strong footshock	Effect nullified by BDNF impaired function mutation val66met
Zhao et al., 2023	Male, Mice, C57BL/6J, 2 months (Adult) or 10 months (middle-aged)	10 mg/kg IP, 1 h before FC and each extinction session		Within-session extinction	Enhanced on fourth trial in middle-aged mice	Normalized a fear-associated enhancement of hippocampal LTP and PPF
Glavonic et al., 2024	Male, Mice, C57BL/6, Adolescent	10 mg/kg IP, 1 h before each extinction session		Within-session extinction	Impaired	Enhanced BDNF mRNA levels, Akt-mTOR-GluA1 signaling, and c-Fos in the hippocampus and mPFC
				Between-session extinction	Enhanced	
				Long-term fear extinction	Enhanced	

Hyphen (-) indicates no effect of ketamine on fear extinction at the selected dose, route, and timing.

IP, intraperitoneal; FC, fear conditioning; S-D, Sprague-Dawley; mTORC1, mammalian/mechanistic target of rapamycin complex 1; mPFC, medial prefrontal cortex; AMPA, α-Amino-3-hydroxy-5-methyl-4-isoxazolepropionic acid; NBQX, 2,3-dioxo-6-nitro-7-sulfamoyl-benzo[f]quinoxaline; BDNF, brain-derived neurotrophic factor; mRNA, messenger RNA; CFC, contextual fear conditioning; LTP, long-term potentiation; PPF, paired-pulse facilitation; CA1, cornu ammonis 1; PL-PFC, prelimbic prefrontal cortex; CS-, conditioned stimulus not paired with aversive stimulus; val66met, valine → methionine substitution at codon 66 of BDNF gene; Akt, protein kinase B; GluA1, AMPA glutamate receptor 1.

Two of the studies that found enhanced fear extinction after ketamine utilized multiple time points of administration, and demonstrated that ketamine has different effects on fear extinction based on the timing of treatment. For instance, ketamine (10 mg/kg, IP) enhanced early fear memory extinction learning in rats when given immediately after FC and also 1 h before extinction training, which took place after 10 days after fear conditioning, but not 1 h before FC (Kulyk, 2017). In contrast, a higher dose ketamine (30 mg/kg, IP), given 1 week before contextual fear conditioning (CFC), reduced freezing in the first fear extinction trial in mice (McGowan et al., 2017). Interestingly, this effect was not observed when ketamine was administered at different time points such as 1 month, 1 day, or 1 h before the CFC, or between CFC and fear extinction (McGowan et al., 2017). In addition to the differences in timing of administration, these studies vary in ketamine dosages, and the rodent species used, so it is plausible that other factors may have contributed to heterogeneous effects of ketamine on fear extinction.

Moreover, the Kulyk et al. study used cued fear conditioning and fear extinction, whereas the McGowan et al. study used CFC and fear extinction. Neural circuitry involved in different aspects of fear extinction has been reported previously (Herry et al., 2010). As fear extinction learning is especially dependent upon the fear-associated context, many studies indicated the contribution of hippocampal regions for contextual fear extinction (Bouton, 2004; Ji and Maren, 2007). Moreover, when it comes to cued fear and contextual fear learning, the amygdala plays a major role in fear memory. For example, lesions of the hippocampus only interfered with contextual fear whereas lesions of amygdala interfered both contextual and cued fear indicating the involvement in both simplistic and complex conditioned stimuli in fear learning (Phillips and LeDoux, 1992). It is important to note that sub-regions of amygdala and hippocampus interact with each other to modulate fear extinction processes. Thus, the underlying neural circuits and fear extinction mechanisms may also differ between these studies; the hippocampus is involved more prominently in contextual than cued FC, as shown in studies where hippocampal-lesioned rats can still acquire cued fear conditioning (Phillips and LeDoux, 1992).

Glutamatergic AMPA receptors and NMDA receptors are vital to ketamine's mechanism of fear memory. Girgenti et al. (2017) found that ketamine (10 mg/kg, IP), given 24 h after fear conditioning, enhanced fear extinction on the second of three extinction days. Ketamine reduced freezing in a combined cue and contextual fear renewal test 1 week later, which indicates long-term fear extinction retention. In this study, intra-mPFC infusion of the AMPA receptor antagonist 2,3-dioxo-6-nitro-7-sulfamoyl-benzo[f]quinoxaline (NBQX) partially blocked the effects of ketamine on enhanced fear extinction on day 2 (Girgenti et al., 2017). The downstream effects of glutamatergic release by ketamine include synaptic protein synthesis, which includes the synthesis of AMPA receptor subunits (Aleksandrova et al., 2017). Glavonic et al. (2024) administered ketamine (10 mg/kg, IP) 1 h before each fear extinction session with four sessions, each with five trials, separated by 24 h. In this study, mice experienced between-session fear extinction as evidenced by reduced freezing in the first trial of session 4 compared to the last trial of session 3. Ketamine also increased long-term fear extinction, with reduced freezing in trial 1 between the first and fourth sessions. On day 4, ketamine upregulated expression of AMPA receptor subunit GluA1 and NMDA receptor subunit GluN2A levels in the hippocampus of mice (Glavonic et al., 2024). Therefore, both the initial activation and downstream synthesis of AMPA and NMDA glutamate receptors are implicated in the effects of ketamine on fear extinction.

Three studies demonstrated that ketamine's effect on fear extinction is mediated by BDNF. Mirroring McGowan et al., Ryan et al. (2022) utilized a prophylactic dose of ketamine (30 mg/kg, IP) 1 week before fear conditioning. Mice received randomly assigned presentations of two distinct auditory tones, one paired with electric footshock (CS+) and the other unpaired (CS–). Mice receiving a strong footshock (1 mA) froze equally to CS+ and CS–, indicating fear generalization. Ketamine reduced freezing to CS– across extinction sessions, which was abolished in mice with the BDNF impaired-function mutation val66met (Ryan et al., 2022). Similarly, ketamine enhanced activity in the ventral cornu ammonis 1(vCA1) of the hippocampus in the post-cue interval after CS– compared to CS+, indicating updating of CS+ and CS– memory; this was not observed in val66met mice (Ryan et al., 2022). BDNF may contribute to ketamine's prophylactic efficacy by enhancing strength and connectivity in critical fear regions such as the mPFC and hippocampus. Ju et al. (2017) compared higher, short-term (5, 10, 20 mg/kg, IP, 2h before extinction sessions on days 15 and 16) and lower, long-term (0.625, 1.25, or 2.5 mg/kg, IP, for 22 consecutive days) doses of ketamine on fear extinction. Spontaneous recovery and fear renewal were assessed on day 23. Long-term ketamine administration did not change within-session fear extinction, but did reduce fear in subsequent spontaneous recovery and fear renewal sessions, suggesting an enhancement of fear extinction memory as evidenced by attenuated fear relapse (Ju et al., 2017). The fear conditioning paradigm led to several changes in the BDNF-mediated pathways in the hippocampus and mPFC, such as increased DNA methyltransferase levels, downregulated BDNF mRNA expression, and increased BDNF gene DNA methylation, which were reversed by long-term ketamine and extinction training (Ju et al., 2017). Similarly, ketamine (10 mg/kg, IP) administered 1 h before four extinction sessions enhanced fear extinction memory between the sessions and upregulated mRNA expression of BDNF exon IV in the hippocampus and mPFC of fear-extinguished mice (Glavonic et al., 2024). Taken together, a growing body of literature supports the significance of BDNF in ketamine's effects on fear extinction.

In addition to BDNF, several studies examined the role of its downstream target, the mTOR pathway, in ketamine's effects on fear extinction. The mTOR pathway is regulated upstream by protein kinase B (Akt) and extracellular signal-regulated kinase (ERK), and has downstream targets including 70S6K (Zanos and Gould, 2018). Glavonic et al. (2024) reported that ketamine enhanced between-session fear extinction and increased phosphorylation of mTOR and Akt in the hippocampus. In a study by Girgenti et al. (2017), ketamine increased phosphorylated Akt, ERK, and 70S6K levels in the mPFC. In addition, blockade of the mTOR complex 1 (mTORC1) pathway via intra-mPFC infusion of rapamycin abolished ketamine's effects on fear extinction (Girgenti et al., 2017). Therefore, the mTOR pathway may also be involved in the effects of ketamine on fear memory extinction. A diagram of potential molecular mechanisms of ketamine on fear memory on a synaptic level including AMPA and NMDA glutamate receptors, BDNF, mTOR pathways is shown in Figure 1.

The synaptic protein synthesis resulting from mTOR pathway activation leads to modulation of synaptic plasticity, which influences ketamine's effect on fear extinction. Two studies examined the effects of ketamine on fear extinction and LTP as a molecular mechanism (Wei et al., 2020; Zhao et al., 2023). Both studies utilized a CFC paradigm with four fear extinction sessions, spaced 24 h apart, with ketamine (0 or 10 mg/kg, IP) administered 1 h before each extinction session. Specifically, ketamine administration enhanced fear extinction on days 3 and 4 in adolescent mice, and *in vitro* ketamine treatment (100 μM) reduced hippocampal CA1 LTP in fear-extinguished mice (Wei et al., 2020). Additionally, 10-month-old mice showed impaired fear extinction compared to 2-month-old mice. Follow-up experiments in 10-month-old mice revealed that ketamine (10 mg/kg, IP) enhanced fear extinction and reduced LTP in the CA1 compared to saline on the 4th day of fear extinction (Zhao et al., 2023). Interestingly, freezing and LTP magnitude were positively associated regardless of age or drug treatment in mice (Zhao et al., 2023). Sala et al. (2022) administered ketamine (10 mg/kg, IP) 6 h after footshock stress and found enhanced fear extinction on days 3 and 4 in rats. In this study, footshock-stressed rats had higher peak amplitude of spontaneous excitatory post-synaptic current (sEPSC) in the prelimbic PFC (PL-PFC), which was normalized by ketamine administration (Sala et al., 2022). Taken together, these studies suggest that impaired fear extinction may be associated with abnormal neuronal excitability, and ketamine enhances extinction while normalizing signaling related to learning, memory, and synaptic plasticity.

### Ketamine impaired fear memory extinction

Two studies reported that ketamine administration impaired fear memory extinction in rodents, as summarized in Table 2. The route of administration (IV vs. IP) and doses of ketamine administration were different between the studies. Both studies used subanesthetic doses of ketamine, although the dosing of the IP ketamine was higher than most other studies included in this review. The timing of ketamine administration also differed between the studies; the IP study administered ketamine 30 minutes before fear conditioning or fear memory extinction, or immediately after fear memory extinction (Clifton et al., 2018). In contrast, the IV ketamine study administered ketamine immediately following fear conditioning (Radford et al., 2022).

**Table 2:** Summary of literature that demonstrates worsened fear extinction with ketamine.

**Study**	**Sex, species, strain, age**	**Dose, route, timing**		**Type of fear/extinction**	**Effect on fear/extinction**	**Notes**
Clifton et al., 2018	Male, rats, Lister-Hooded, adult	8 or 25 mg/kg, IP	30 min before extinction training	Between-session extinction	Impaired	Study controlled for context-sensitive effects of ketamine
		25 mg/kg, IP	Immediately after extinction	-	-	
Radford et al., 2022	Female, rats, S-D, 9 weeks	2, 10, or 20 mg/kg, IV infusion, given immediately following FC		Extinction retention	Impaired	IV ketamine increased plasma CORT and PROG levels
				Long-term fear expression	Enhanced	

Hyphen (-) indicates no effect of ketamine on fear extinction at the selected dose, route, and timing.

mg/kg, milligram/kilogram; IP, intraperitoneal; S-D, Sprague-Dawley; IV, intravenous; FC, fear conditioning; CORT, corticosterone; PROG, progesterone.

Clifton et al. described the impact of ketamine on CFC. Male Lister-Hooded rats were fear-conditioned using electric footshock, followed by tests on contextual fear memory consolidation and extinction. Ketamine (8 or 25 mg/kg, IP) administered prior to extinction training had no effects on within-session fear extinction. However, ketamine administered before fear extinction may have impaired the consolidation of extinction memory (between-session extinction) as freezing was increased in subsequent fear memory recall trials (Clifton et al., 2018). Interestingly, the authors hypothesized that there may be state-dependent effects of ketamine when given prior to fear memory consolidation, such that animals are able to recall events when the internal context (e.g., presence or lack of ketamine) matches the state that they acquired the memory (i.e. before CFC). The authors considered that this may have confounded ketamine's effects on extinction retention at the 25 mg/kg dose, where rats receiving ketamine before CFC and extinction had enhanced freezing after recall 1 (ketamine) but not recall 2 (saline). However, at 8 mg/kg, ketamine given 30 min before extinction training impaired extinction retention in both recall trials regardless of drug condition (ketamine before recall 1, saline before recall 2), suggesting ketamine's impairment of extinction consolidation occurred irrespective of state-dependency. In summary, ketamine impaired the consolidation of extinction memory, which may have been influenced by state-dependency depending on the dosage of ketamine.

Radford et al. administered an IV ketamine infusion (0, 2, 10, and 20 mg/kg) over a 2-h period to adult female Sprague-Dawley rats immediately after auditory fear conditioning. They noted enhanced cued fear memory expression, impaired extinction retention as freezing peaked in the first block of cue test 2, and increased cued and contextual fear renewal (Radford et al., 2022). Additionally, IV ketamine infusion dose-dependently increased plasma stress hormone levels, corticosterone (CORT) and progesterone (PROG), in female rats. Regional brain glucose metabolism after IV ketamine infusion was also assessed using ^18^F-fluorodeoxyglucose positron emission tomography (^18^F-FDG-PET). Ketamine altered regional glucose metabolism compared to the saline control, increasing levels in the cortex and decreasing the levels in the thalamus, hypothalamus, and midbrain. Notably, this is the only study that used female rats and further studies are necessary to validate the findings from the study. Nevertheless, overall findings on fear extinction and plasma stress hormone levels are similar to the previous studies that found impaired fear extinction and/or elevated stress hormones when subanesthetic doses of IV ketamine were infused immediately after fear learning in male rats (Radford et al., 2018, 2020b). Thus, these studies suggest that route and timing of ketamine administration may be a crucial factor for modulating stress-related outcomes.

### No effects or mixed effects of ketamine on fear memory extinction

Four studies reported no effects or mixed effects of ketamine on fear memory extinction. The doses and route of ketamine administration were variable: one study used an intramuscular (IM)/IP route, dividing 250 mg/kg into four doses (100 mg/kg IM, 3 x 50 mg/kg IP) over 4 h; one study utilized only an IP route, administering 1 or 10 mg/kg of ketamine; and two studies utilized a subanesthetic IV infusion in rodents. The timing of ketamine administration also varied across these studies, as summarized in Table 3.

**Table 3:** Summary of literature that demonstrates mixed or no effects of ketamine on fear extinction.

**Study**	**Sex, species, strain, age**	**Dose, route, timing**	**Type of fear/extinction**	**Effect on fear/extinction**	**Notes**
Groeber Travis et al., 2015	Male, rats, S-D, adult	250 mg/kg divided into 4 doses over 4 h (100 mg/kg IM, 3 × 50 mg/kg IP after FC and/or extinction)	Within-session extinction	No effect	Study used an operant/appetitive model for effects of fear memory extinction behaviors; ketamine reduced appetitive behavior unrelated to extinction
Radford et al., 2018	Male, rats, S-D, adult	2, 10, 20 mg/kg IV, immediately after FC	Within-session extinction Long-term fear expression	Impaired with 10 mg/kg IV Enhanced with 10 mg/kg IV	Inverted U-shape dose-response curve for IV ketamine: 10 mg/kg impaired fear memory, whereas 2 and 20 mg/kg did not
		10 mg/kg IP, immediately after FC	Within-session extinction Long-term fear expression	Enhanced Reduced	
Radford et al., 2020	Male, rats, S-D, 9 weeks	10 mg/kg IV infusion, immediately after FC	Within-session extinction, between-session extinction, long-term fear expression	No effect	Correlation between elevated stress hormones (CORT and PROG) and cued fear renewal
Gokalp et al., 2024	Male, rats, Wistar, 3–6 months	1 or 10 mg/kg IP, 30 min before extinction I	Within-session extinction	No effect of ketamine alone	Interaction between MTEP (mGluR5 antagonist) and low-dose ketamine (1 mg/kg) on extinction I

S-D, Sprague-Dawley; mg/kg, milligram/kilogram; IM, intramuscular; IP, intraperitoneal; IV, intravenous; FC, fear conditioning; PTSD, posttraumatic stress disorder; CORT, corticosterone; PROG, progesterone; MTEP, 3-((2-Methyl-4-thiazolyl)ethynyl)pyridine; mGluR5, metabotropic glutamate receptor 5.

Groeber Travis et al. (2015) examined the effects of ketamine on fear memory extinction using an operant conditioning paradigm. Food-restricted Sprague-Dawley rats were trained in operant conditioning chambers to press a lever to self-administer food pellets via a variable interval (VI) schedule. Following this training, animals underwent FC in the chamber resembling the operant VI schedule chamber (house lights, stimulus lights, etc.) but with a metal grid allowing for an electric footshock after an audio-visual cue. The VI operant conditioning took place over 12 experimental days, with every other VI session having an extinction paradigm. Rats received four consecutive doses of ketamine (100 mg/kg IM, 3 × 50 mg/kg IP, each 1 h apart) or saline after fear conditioning and/or fear extinction sessions. Reductions in overall lever presses for food pellets (response rate) and reduction after the CS (suppression index) were measured. No effects were noted on fear memory extinction using this paradigm, with no changes in suppression index on extinction days, but the overall response rate was reduced on days following ketamine administration (Groeber Travis et al., 2015). This study suggests that ketamine produced symptoms that were potentially maladaptive (i.e. impaired reward learning) but not related to fear learning.

Gokalp and Unal (2024) administered ketamine prior to two fear extinction sessions, termed extinction I and extinction II. Rats received either a low (1 mg/kg, IP) or high (10 mg/kg, IP) dose of ketamine, 30 min before fear extinction. Ketamine alone did not affect freezing behaviors in fear conditioning or extinction sessions. However, in rats that received the metabotropic glutamate receptor 5 (mGluR5) antagonist MTEP (1.25 mg/kg, IP) 10 min before low-dose ketamine injection, MTEP and ketamine synergistically reduced freezing in fear extinction I (Gokalp and Unal, 2024). Thus, it is possible that ketamine alone may not affect fear extinction, but when combined with other drugs, ketamine may enhance fear extinction.

As in previous studies, Radford et al. (2020b) administered IV ketamine (10 mg/kg, 2-h infusion) to adult male Sprague-Dawley rats immediately after FC on day 0. Extinction tests took place 1 week later, with extinction acquisition on day 7, extinction recall on day 8, and cued and contextual fear renewal on day 9. No significant differences were found between ketamine and control animals in fear extinction (Radford et al., 2020b). However, the authors found the association between elevated plasma stress hormone levels (CORT and PROG) following ketamine infusion and cued fear renewal, suggesting that individual differences in stress responses may influence the vulnerability for abnormal fear memory.

In a separate study, Radford et al. (2018) found that IV ketamine (0, 2, 10, or 20 mg/kg, 2 h infusion) administered immediately after auditory fear conditioning produced dose-dependent effects on fear memory extinction and fear memory recall. A moderate dose IV ketamine (10 mg/kg) infusion impaired fear extinction more so than lower or higher doses, indicating an inverted U-shape dose-response curve. Fear retrieval was normal as evidenced by normal freezing in the initial blocks of cue test 1; IV ketamine specifically impaired fear extinction learning, as freezing was heightened in the later blocks of the session. Interestingly, the same dose of ketamine (10 mg/kg), when injected via an IP route, produced opposite effects on fear extinction in male rats. IP ketamine increased fear extinction learning, lowering total freezing throughout the session starting from the second block (Radford et al., 2018). Kulyk (2017) also found enhanced extinction at 10 mg/kg IP ketamine immediately post-FC, but through impaired fear consolidation rather than enhanced extinction learning. The *in vivo* glucose metabolism by ^18^F-FDG-PET imaging of the brain indicated that IV ketamine infusion altered regional energy utilization such as the hippocampus, amygdala, and hypothalamus, suggesting the importance of those regions in fear memory and ketamine's effects (Radford et al., 2018). Of note, this study highlights the importance of the route (IV vs. IP) and duration (continuous vs. bolus administration) of ketamine on fear memory, as the same dose of ketamine (10 mg/kg) can produce heterogeneous effects on fear memory in rats.

## Discussion

The current review indicates that the effects of ketamine on fear extinction are dependent upon several factors such as the dosages, timing, and route of ketamine administration (Choi et al., 2020; Radford et al., 2018). Overall, subanesthetic doses of ketamine injection using an IP route appear to facilitate fear extinction when given after the fear conditioning or before fear extinction. IP ketamine injection was associated with an increase in fear extinction in the majority of published articles. Unlike IV ketamine, which can reach peak plasma levels in as little as 1 min after bolus administration and a half-life of around 2 h (Marietta et al., 1976; Le Nedelec et al., 2018), IP ketamine has a delayed and lower peak due to first-pass metabolism via the liver (Nguyen et al., 2022). The steady-state plasma levels of ketamine from IV infusion may result in the enhancement of noradrenergic release from the basolateral amygdala (BLA). Increases in noradrenergic releases from the BLA are associated with stronger aversive memory formation (Roozendaal et al., 2004) and may explain why IV ketamine infusion was associated with impaired, rather than facilitated, fear extinction (McIntyre et al., 2002; Morena et al., 2021). Additionally, there may be a positive association between fear behavior and plasma stress hormone levels, regardless of the overall findings of ketamine on fear extinction (Radford et al., 2022, 2020b). Thus, the route of ketamine administration may be crucial due to different bioavailability and duration of drug action which can have consequences such as amplified stress hormonal alterations.

The timing of ketamine administration is also important for fear extinction. Impaired fear extinction by ketamine commonly resulted from a single IV ketamine infusion administered immediately after fear conditioning (Radford et al., 2022, 2018). This may be due to paradoxical strengthening of fear memory consolidation with analgesic doses of continuous IV ketamine infusion (10–20 mg/kg, 2 h) given immediately after fear learning. During the IV ketamine infusion period, animals are calm and slightly sedated due to continuous infusion of ketamine through indwelling jugular venous catheters. Though locomotor activity levels are lower due to analgesic and sedative effects, plasma stress hormone levels are significantly elevated in those animals receiving a continuous IV ketamine infusion (Radford et al., 2018, 2020b). Thus, the HPA axis stimulation during emotional episodic memory consolidation may enhance that type of memory as previously reported in various types of learning and memory studies (Sandi and Pinelo-Nava, 2007; de Kloet et al., 1999; McGaugh, 2000; McGaugh and Roozendaal, 2002). Although this could be context-dependent (e.g. mildly stressed vs. traumatically stressed), elevated stress hormone levels by IV ketamine infusion during the fear memory consolidation period may contribute to impaired fear extinction in the animals.

Fear memory consolidation is a process where short-term memory is potentiated into long-term memory over a several-hour time window (Schafe and LeDoux, 2000; de Quervain et al., 2017; Johansen et al., 2011). When ketamine was administered after the fear memory consolidation time window, ketamine enhanced fear memory extinction as shown in Table 1. In those studies, ketamine's effects on the HPA axis and sympathetic nervous system may be less crucial due to the time window of post-consolidation. Thus, it is possible that ketamine produces differential effects on fear extinction depending on the timing of administration: enhanced fear extinction when ketamine is administered outside of the memory consolidation period or worsened/ineffective fear extinction when ketamine is administered within the memory consolidation period. This may apply to other types of fear learning as well; in a study of mice undergoing FC 24 h before fear generalization, ketamine (30 mg/kg, IP) administered 22 h after, but not a week before, an hour before, or immediately after FC, alleviated fear generalization, an effect that remained stable for 2 weeks (Asim et al., 2020). Similarly, an infusion of ketamine (15 μg/μL) into the nucleus accumbens (NAc) 22 h after FC alleviated fear generalization for 2 weeks (Asim et al., 2022). This idea has significant implications for clinical research and practice, as acute post-trauma ketamine administration may worsen the development of PTSD symptoms (Melcer et al., 2022; Schonenberg et al., 2008; Brodeur et al., 2020; Highland et al., 2020) while ketamine administration combined with CBT and PE to PTSD patients may improve PTSD symptoms (Pradhan et al., 2018, 2017; Shiroma et al., 2020; Harpaz-Rotem, 2022).

Ketamine was able to enhance fear memory when given shortly after FC due to factors such as HPA axis activation, which can enhance fear memory consolidation in emotional learning (Radford et al., 2022, 2020b; de Quervain et al., 2017). Another explanation for enhanced fear memory during consolidation is “retrograde facilitation”, the enhancement of memory encoded prior to drug administration, caused by various drugs including alcohol, midazolam, and propofol (Hauer et al., 2011; Hewitt et al., 1996; Parker et al., 1980; Reder et al., 2007). In the same vein, ketamine may also enhance fear memory extinction if given after fear extinction learning. Studies on D-cycloserine (DCS), a partial NMDA receptor agonist, have found that DCS is most efficacious when given shortly before or after exposure therapy (Grillon, 2009). Administration after a session may be particularly helpful to selectively strengthen memories and associations of productive therapy sessions, and because DCS peaks in plasma 4–8 h after administration, which aligns with the height of post-consolidation extinction learning (Davis, 2011; Norberg et al., 2008). These data indicate the paradoxical effects of DCS and ketamine, and suggest that the timing of drug administration on fear extinction is critical.

Studies testing the direct interplay between memory reconsolidation and memory extinction provide evidence supporting the critical effect of timing on extinction and reconsolidation and the distinct cellular mechanisms and circuits underlying them (Ferrara et al., 2023). In both preclinical and clinical studies, memory retrieval followed by extinction training enhanced fear reduction and reduced relapse to fear, suggesting disruption of the original fear memory via disruption of the memory reconsolidation window (Monfils et al., 2009; Schiller et al., 2010). The delineation between disruption of memory reconsolidation and memory extinction has revealed that a few unreinforced auditory CS presentations (*n* < 5) triggered reconsolidation, an effect dependent on AMPA glutamate receptor modifications in the amygdala (Ferrara et al., 2023; Clem and Huganir, 2010). In contrast, many more CS presentations (*n* = 40) initiated fear extinction, an effect characterized by decreased phosphorylation of cAMP response element-binding protein (CREB), a transcription factor involved in learning and memory (Ferrara et al., 2023). While limited, these emerging data suggest distinct mechanisms and neural circuits underlying the modulation of memory reconsolidation and memory extinction that may be independently targeted by ketamine administration with specific timing, route, and dosages.

These data raise important questions on the mechanism of action and the utility of ketamine for PTSD treatment. In contrast, disruption of reconsolidation of an established extinction memory could occur in a separate time window when ketamine is administered following fear recall or testing of extinguished fear memory. These emerging results suggest that both fear memory consolidation and reconsolidation are time-sensitive processes that depend on NMDA glutamate receptor-mediated calcium influx in the postsynaptic neurons as well as phosphorylated ERK/mitogen-activated protein kinase (MAPK)-mediated signaling in the brain (Bergstrom et al., 2013; Duvarci et al., 2005; Wang et al., 2009). As described in Figure 1, ketamine can produce paradoxical increase in glutamate release by inhibiting inhibitory GABA interneurons (by blocking NMDA receptors) that control excitatory glutamatergic neurons in the PFC as reported previously (Moghaddam et al., 1997). Thus, increased glutamate release stimulates post-synaptic neurons by increasing calcium influx through NMDA receptors and intracellular signaling pathways such as ERK/MAPK in the frontal cortex, which acts on mTOR and other pathways to influence downstream synaptic plasticity and synaptic protein synthesis. This may overall contribute to proposed synaptic plasticity in the brain and strengthening fear memory consolidation by post-fear learning ketamine administration.

The time window for memory consolidation and reconsolidation is ~3 h with the NMDA receptor-mediated window occurring at the beginning of the intracellular signaling cascade (Raut et al., 2022a; Duvarci et al., 2005; Wang et al., 2009). Emerging preclinical and clinical studies have demonstrated that ketamine and the NMDA receptor non-competitive antagonist MK-801 can interact with memory reconsolidation mechanisms to reduce neural activity in the amygdala (Duek et al., 2023) as well as fear-related behaviors in animals (Li et al., 2024; Tiunova et al., 2024) and PTSD symptoms in humans (Duek et al., 2023). Subanesthetic doses of ketamine may interact with traumatic memory consolidation when administered for pain management in the peri-trauma period. Alternatively, ketamine may be administered weeks to months after the initial trauma in the clinical settings when ketamine has the opportunity to influence the reconsolidation of the trauma memory and/or the consolidation of newly formed fear extinction memory. Ketamine may interact with a newly consolidated prefrontal-cortex dependent fear extinction memory. However, in order to interfere with the reconsolidation of memory, consolidated memory must be brought into a labile state first, and historical and previously consolidated long-term traumatic memories may not always be amenable to manipulate with ketamine or other drugs such as propranolol (Raut et al., 2022a).

This review also reflects the significance of different dosages of ketamine on fear extinction. In rodent studies, subanesthetic doses of ketamine can be determined based on behavioral deficits such as loss of righting reflex, ataxia, and agitation [<60 mg/kg for the IP route and <10 mg/kg for the IV route as previously reported (Youth et al., 1973; Cohen et al., 1973)]. Following IV bolus ketamine administration to rats, peak brain levels are achieved less than a minute with preferential distribution to the cortical regions (Cohen et al., 1973). Subanesthetic doses of ketamine can still produce dose-dependent effects on locomotor activity, analgesia, and dissociative stereotypy in animals (Danysz et al., 1994; Imre et al., 2006; Radford et al., 2017). The overall findings in the literature favor low-dose ketamine (<10 mg/kg) for enhancing fear extinction. As previously mentioned, IV ketamine infusion results in a higher plasma steady-state level, which may impact the effects of ketamine on fear memory extinction. Thus, these findings may potentially translate to clinical practice, favoring sufficient analgesic doses without severe dissociative side-effects in traumatically injured patients. Some studies examine a strategy of combining sub-effective ketamine with other interventions to minimize side-effects of ketamine and other treatments. A sub-effective 1 mg/kg ketamine injection paired with 8-tone extinction was able to synergistically reverse stress-induced attentional set-shifting task (AST) deficits to the same extent as either 3–10 mg/kg ketamine or 16-tone extinction (Paredes et al., 2022). Similarly, a sub-effective dose of ketamine (1 mg/kg) combined with MTEP, an mGluR5 antagonist, produced synergistic effects on fear extinction learning (Gokalp and Unal, 2024).

Ketamine may not affect all metrics of fear extinction in the same manner. For example, ketamine increased between-session fear extinction, demonstrating that ketamine administration is associated with overall reduction in fear memory, while ketamine reduced within-session fear extinction in the same study (Glavonic et al., 2024). This may simply reflect the increased fear response at the start of the extinction session in the control animals (i.e., more room for improvement compared to the ketamine-treated animals). Additionally, the authors suggest that ketamine may also enhance long-term fear extinction learning, which reduced within-session freezing changes (Glavonic et al., 2024). For this reason, between-session fear extinction should be measured by differences in initial freezing across the multiple sessions (Wotjak, 2019) in order to standardize fear extinction paradigms.

There are other sources of variations across fear extinction paradigms. This includes the number of extinction sessions, ranging from one to eight or more (McGowan et al., 2017), which may affect drug response to fear extinction learning (Grillon, 2009). In the case of multiple fear extinction sessions, some studies define them as extinction training (Glavonic et al., 2024; Girgenti et al., 2017; Wei et al., 2020; Zhao et al., 2023) whereas others define the first extinction session as training or fear retrieval and the second as an extinction retrieval or retention (Kulyk, 2017; Radford et al., 2022, 2018, 2020b). Such differences in definitions and terminology can cause confusion and hinder comparability between the studies. In general, fear extinction sessions are followed by retention tests which reintroduce the CS or context in a reduced capacity (e.g. frequency, duration, and intensity) so that it cannot trigger fear extinction learning again. Other paradigms measure extinction retention with tests specifically intended to trigger a return of fear, such as spontaneous recovery, reinstatement, or renewal, which are considered more robust measures of fear extinction retention (Wotjak, 2019). Spontaneous recovery, the return of fear after an extended period of time, represents another important variable: the timing of tests within the paradigm. An IV ketamine infusion administered after fear conditioning impaired fear extinction when measured 2 and 3 days later (Radford et al., 2022, 2018), but not a week later (Radford et al., 2020b), suggesting that ketamine's effects on fear memory may be short-lasting. While standardizing fear conditioning paradigms may not be practical, it is important to be aware of methodological differences when critically examining the previous literature.

## Limitations and future directions

In light of the timing of ketamine administration, future research should consider administering ketamine shortly before fear extinction to evaluate its potentially beneficial effects on PTSD. This resembles the use of ketamine as an adjunct therapy to psychotherapy such as PE and CPT, which has shown some promising results in PTSD (Pradhan et al., 2018, 2017; Shiroma et al., 2020; Harpaz-Rotem, 2022). The current review of the preclinical literature indicates that ketamine may exert either adverse or beneficial effects on fear memory extinction depending on the timing, route, and dosages of ketamine administration.

A small scale, open-label, proof of concept study was performed using standardized PE therapy with repeated ketamine administration to 10 veterans with PTSD (Shiroma et al., 2020). Moderate to severe PTSD patients based on Clinician Administered PTSD Scale for DSM-5 (CAPS-5) and DSM-5 diagnoses received IV ketamine infusion (0.5 mg/kg) 1 day prior to weekly PE for 3 weeks. PE therapy was continued up until 10 weeks. They found that ketamine as an adjunct pharmacotherapy combined with extinction-based therapy (e.g. PE) may benefit PTSD treatment. As a follow-up of the previous pilot study (Shiroma et al., 2020), the same group is conducting a double-blind, randomized, single-site clinical trial to determine the efficacy, safety, and tolerability of IV ketamine infusion to enhance PE therapy among veterans with PTSD (Shiroma et al., 2024). This study will compare IV ketamine treatment vs. active placebo (midazolam) adjunct to PE therapy among 100 veterans with PTSD. Pharmacological phase will start simultaneously with PE session 1, with ketamine or placebo administration one day prior to the PE session for the first 3 weeks. After 10 PE sessions are completed, patients will be assessed for PTSD symptoms during a 3-month follow-up period. Other psychiatric symptoms including depression and anxiety disorders, as well as safety and tolerability of ketamine-enhanced PE therapy will be assessed.

One difficulty in applying this strategy to clinical practice is that many patients may have received analgesic doses of ketamine to treat traumatic injury. In the preclinical studies, IV ketamine infusion immediately after fear conditioning enhanced fear memory (Radford et al., 2022, 2018). Moreover, ketamine may produce state-dependent effects such that in patients receiving ketamine in the peri-trauma period, ketamine may reintroduce those traumatic memories at the time of fear extinction session (Clifton et al., 2018). However, these effects were dose-dependent, present at higher dose (25 mg/kg), but not at lower dose (8 mg/kg) which is more relevant to the clinical doses. Ketamine is an excellent trauma analgesic due to its high safety ceiling such as hemodynamic and respiratory stability. Thus, it is worthwhile to examine the precise time course and mechanisms of ketamine on fear memory extinction to weigh the risks and benefits in its use for treatment of PTSD.

Sex is another important factor to be considered in ketamine research. Despite the fact that PTSD rates are higher in women than men (Christiansen and Berke, 2020), only one preclinical study in this review investigated the effects of ketamine on fear extinction using female rats. A wide range of ketamine doses (2, 10, and 20 mg/kg, IV) impaired fear extinction in female rats (Radford et al., 2022), while only one dose (10 mg/kg) was effective in the previous study with male rats (Radford et al., 2018). Alterations in behaviors between males and females have been noted in ketamine studies with both IV and IP routes which were partially mediated by circulating gonadal hormones (McDougall et al., 2019; Radford et al., 2020a). Additionally, pharmacokinetics and pharmacodynamics of ketamine are known to be different between male and female rats, with female rats exhibiting slower drug clearance and therefore, longer duration of action of ketamine (Saland and Kabbaj, 2018). This could contribute to enhanced behavioral sensitivity to ketamine in females and may explain why IV ketamine is more potent at a wider range of doses in female rats compared to male rats. Thus, more female rodents should be included in future studies as sex differences are important and the influence of gonadal hormones between male and females may contribute to heterogeneous effects of ketamine on PTSD.

Several studies in this review included other types of behavioral assays or paradigms while examining the effects of ketamine on fear extinction. PTSD is not a psychiatric disorder of only exaggerated fear responses, but also includes aspects of anhedonia, lack of appetite, loss of motivation, anxiety, social isolation, and risk-taking behaviors (American Psychiatric Association, 2022). For instance, ketamine may affect both fear memory extinction and depression-like behaviors based on the forced swim test in the same study (Wei et al., 2020). Another study also examined open field behaviors and pain thresholds along with extinction at the same time point and found no significant changes in those additional measures (Zhao et al., 2023). Altered fear extinction may be associated with anxiety behaviors as shown by improved performance in the open field test and elevated plus maze (Ju et al., 2017). Sala et al. (2022) reported no relationship between fear behavior and anhedonia based on the sucrose preference test as ketamine did not alter sucrose intake, and Groeber Travis et al. (2015) demonstrated decreased appetitive behavior which was decreased by ketamine but not related to fear extinction. Low-dose ketamine (1 mg/kg) produced anxiolytic effects based on increased open arm time on the elevated plus maze, whereas a high dose (10 mg/kg) produced antidepressant effects based on decreased immobility time in the forced swim test, although ketamine *per se* did not affect fear extinction (Gokalp and Unal, 2024). Taken together, though fear conditioning is a valuable model of fear-related aspects of PTSD, emphasis on its use alone may obscure the wide array of other symptoms that are associated with PTSD in humans. Thus, models that encompass various aspects of PTSD besides fear, such as depression, anxiety, and motivation, may allow for a more comprehensive examination of PTSD in preclinical studies.

One of the prominent examples of combining fear conditioning with other models is the single prolonged stress (SPS) paradigm. Animals are exposed to ~3 h combined of restraint, forced swim, and loss of consciousness via ether, followed by a quiescent period of 7 days or longer (Liberzon et al., 1997). A recent review article found 33 studies that examined fear memory extinction after SPS exposure (Ferland-Beckham et al., 2021). One study reported that SPS had no effect on extinction learning but impaired extinction retention in both cued and contextual FC, and SPS enhanced renewal of cued fear (Knox et al., 2012). Additionally, the length of the quiescent period may be crucial because a shorter period between SPS exposure and fear memory testing produced no effects on extinction retention (Knox et al., 2012). The SPS paradigm can be particularly useful to study fear extinction in the context of ketamine, a drug that is known to differentially affect fear memory in stressed and unstressed animals (Juven-Wetzler et al., 2014).

Another line of research on the effects of ketamine on PTSD may include traumatic brain injury (TBI) paradigm, which is a common risk factor for PTSD. Recent studies examined the neurobiological mechanisms of ketamine on fear memory after TBI utilizing a fear memory circuit of the amygdala, the hippocampal CA3, and the hippocampal dentate gyrus (DG), which are implicated in fear generalization and fear discrimination in rodents (McGowan et al., 2024; Cui et al., 2024). Mice with TBI showed contextual fear generalization and ketamine administration recovered fear discrimination as evidenced by increased freezing behavior to an aversive vs. a neutral context, suggesting a retrieval of previously learned fear only in the context where a fearful stimulus was present (McGowan et al., 2024). Given the high comorbidity of TBI and PTSD, more studies examining the effects of ketamine on TBI and PTSD paradigms are warranted. Further, identifying the time course and mechanisms of ketamine on fear extinction following TBI could provide fascinating insights into the neurobiology of TBI and PTSD.

## Conclusions

The current review of preclinical literature supports the hypothesis that ketamine administration may produce beneficial effects on fear memory extinction depending on the route, dosages, and timing of ketamine administration. More studies are warranted to investigate specific roles of those variables in ketamine and fear extinction. Additionally, given sex differences in PTSD and ketamine action, both male and female animals should be tested in clinically-relevant behavioral paradigms to fully understand the complex symptoms of PTSD. While much research is still needed both in preclinical and clinical fields, ketamine may prove to be a useful tool to investigate the molecular and cellular mechanisms of PTSD and traumatic memory.

## Data Availability

The original contributions presented in the study are included in the article/supplementary material, further inquiries can be directed to the corresponding author.
